# Effects of long-term sleep disruption on cognitive function and brain amyloid-β burden: a case-control study

**DOI:** 10.1186/s13195-020-00668-5

**Published:** 2020-08-26

**Authors:** Jana Thomas, Sharon J. Ooms, Lara J. Mentink, Jan Booij, Marcel G. M. Olde Rikkert, Sebastiaan Overeem, Roy P. C. Kessels, Jurgen A. H. R. Claassen

**Affiliations:** 1 Department of Geriatric Medicine, Radboud University Medical Center, 6525 GC Nijmegen, The Netherlands; 2 Donders Institute for Brain, Cognition and Behaviour, 6525 HR Nijmegen, The Netherlands; 3 Radboud Alzheimer Centre, 6525 GA Nijmegen, The Netherlands; 4 Department of Radiology and Nuclear Medicine, Radboud University Medical Center, 6525 GC Nijmegen, The Netherlands; 5 Department of Radiology and Nuclear Medicine, Amsterdam University Medical Centers, Academic Medical Center, 1105 AZ Amsterdam, The Netherlands; 6 Sleep Medicine Centre Kempenhaeghe, 5591 VE Heeze, The Netherlands; 7 Eindhoven University of Technology, 5612 AZ Eindhoven, The Netherlands; 8 Department of Medical Psychology, Radboud University Medical Center, 6525 GA Nijmegen, The Netherlands

**Keywords:** Sleep disruption, Shift work, Alzheimer’s disease, Amyloid-β, Cognitive function

## Abstract

**Background:**

Recent evidence indicates that disrupted sleep could contribute to the development of Alzheimer’s disease by influencing the production and/or clearance of the amyloid-β protein. We set up a case-control study to investigate the association between long-term work-induced sleep disruption, cognitive function, and brain amyloid-β burden.

**Methods:**

Nineteen male maritime pilots (aged 48–60 years) with chronic work-related sleep disruption and a sex-, age-, and education-matched control sample (*n* = 16, aged 50–60 years) with normal sleep completed the study. Primary sleep disorders were ruled out with in-lab polysomnography. Additional sleep measurements were obtained at home using actigraphy, sleep-wake logs, and a single-lead EEG device. Cognitive function was assessed with a neuropsychological test battery, sensitive to early symptomatic Alzheimer’s disease. Brain amyloid-β burden was assessed in maritime pilots using ^18^F-flutemetamol amyloid PET-CT.

**Results:**

Maritime pilots reported significantly worse sleep quality (Pittsburgh Sleep Quality Index (PSQI) = 8.8 ± 2.9) during work weeks, compared to controls (PSQI = 3.2 ± 1.4; 95% CI 0.01 to 2.57; *p* = 0.049). This was confirmed with actigraphy-based sleep efficiency (86% ± 3.8 vs. 89.3% ± 4.3; 95% CI 0.43 to 6.03; *p* = 0.03). Home-EEG recordings showed less total sleep time (TST) and deep sleep time (DST) during work weeks compared to rest weeks (TST 318.56 (250.21–352.93) vs. TST 406.17 (340–425.98); *p* = 0.001; DST 36.75 (32.30–58.58) vs. DST 51.34 (48.37–69.30); *p* = 0.005)). There were no differences in any of the cognitive domains between the groups. For brain amyloid-β levels, mean global cortical standard uptake value ratios of ^18^F-flutemetamol were all in the normal range (1.009 ± 0.059; 95% CI 0.980 to 1.037), confirmed by visual reads.

**Conclusions:**

Capitalizing on the particular work-rest schedule of maritime pilots, this study with a small sample size observed that long-term intermittent sleep disruption had no effects on global brain amyloid-β levels or cognitive function.

## Background

Sleep loss has been associated with increased risk of dementia in later life, specifically dementia caused by Alzheimer’s disease (AD). In a meta-analysis of 27 studies with nearly 70.000 participants, sleep loss—mostly defined as self-reported sleep of < 6 h per night—carried an average relative risk of 1.68 (95% CI 1.45 to 1.86) of developing dementia caused by AD [1]. This finding is relevant, because the etiology of late-onset AD remains unknown and therapeutic options are limited, making sleep a potential target for prevention or treatment of AD [2, 3]. The association between sleep loss and AD could be explained by reverse causality, wherein sleep loss is an early, preclinical manifestation of Alzheimer’s pathology [4–6]. However, the association may also be causal, wherein sleep loss contributes to the development of the disease. The latter hypothesis is based on a small number of animal and human studies that have identified mechanisms that could explain how sleep loss may increase the risk of AD. In mouse models of genetic and sporadic AD, for example, sleep loss increased brain amyloid-β accumulation [7–10]. In humans, we have previously showed that a single night of full sleep deprivation impaired the overnight reduction in CSF amyloid-β, causing 10% higher levels of CSF amyloid-β the next morning [11]. Also in humans, selective disruption of slow wave sleep (SWS), without affecting other sleep stages, led to a comparable overnight difference in CSF amyloid-β [12]. Additionally, acute increases (5%) in PET-amyloid-β levels in the hippocampus and thalamus were observed after a single night of full sleep deprivation [13]. Two mechanisms have been proposed to explain the relationship between sleep loss and amyloid-β accumulation. First, the clearance of soluble toxic waste (including amyloid-β) from the central nervous system, characterized by exchange of interstitial and cerebrospinal fluids through the glymphatic pathway, appears more effective during sleep than in wakefulness [14–18]. Second, the production of amyloid-β may be increased during wakefulness and reduced during sleep (especially SWS) [5, 11, 17, 19, 20]. These findings have led to the hypothesis that long-term sleep loss, through repetitive episodes of amyloid-β accumulation, may contribute to AD. However, evidence from human studies is lacking, and it remains unknown which quantity of sleep loss, both in terms of duration and intensity, would be required to raise the risk of developing dementia due to AD.

The unique work of maritime pilots in the Netherlands offers an opportunity to study the association between long-term sleep disruption and AD risk. Maritime pilots have a work schedule characterized by one week of irregular and unpredictable working hours, leading to reduced and fragmented sleep, followed by a rest week with unrestricted sleep. Their sleep disruptions are caused by external, occupational factors, which reduce the bias of intrinsically caused sleep problems that could represent an early symptom preceding the clinical manifestation of dementia due to AD (i.e., reverse causality). In this group of maritime pilots, we sought to explore the effects of long-term, externally induced sleep disruption on cognitive function and brain amyloid-β burden, as biomarker of AD.

## Methods

### Study design

The SCHIP study (Sleep Cognition Hypothesis In maritime Pilots) is a case-control study in healthy volunteers under the hypothesis that repeated nights of sleep loss may contribute to the risk of dementia due to AD by gradually increasing amyloid-β levels. The study took place between December 2016 and May 2019 and was conducted and reported according to the STROBE guidelines for case-control studies. The timing between measurements was consistent across all participants. Sample size calculations were performed using G*power [21] and published previously [22]. The study protocol was peer-reviewed and published in BMJ Open [22].

### Study population

We included 19 middle-aged (mean age = 53; age range: 48 to 60 years) men from the national organization of Dutch maritime pilots (Nederlandse Loodswezen). The profession of maritime pilots in the Netherlands is almost exclusively (99%) male. Their profession is characterized by sleep disruptions caused by external, occupational factors, wherein every other week is characterized by sleep disruption. We recruited maritime pilots with a work history of an average of 20 years (mean = 19.8; range 10 to 30 years). Details of the study population and their occupation have been described in our methods paper [22] and can be found in the additional information file (see Additional file 1).

**Fig. 1:**
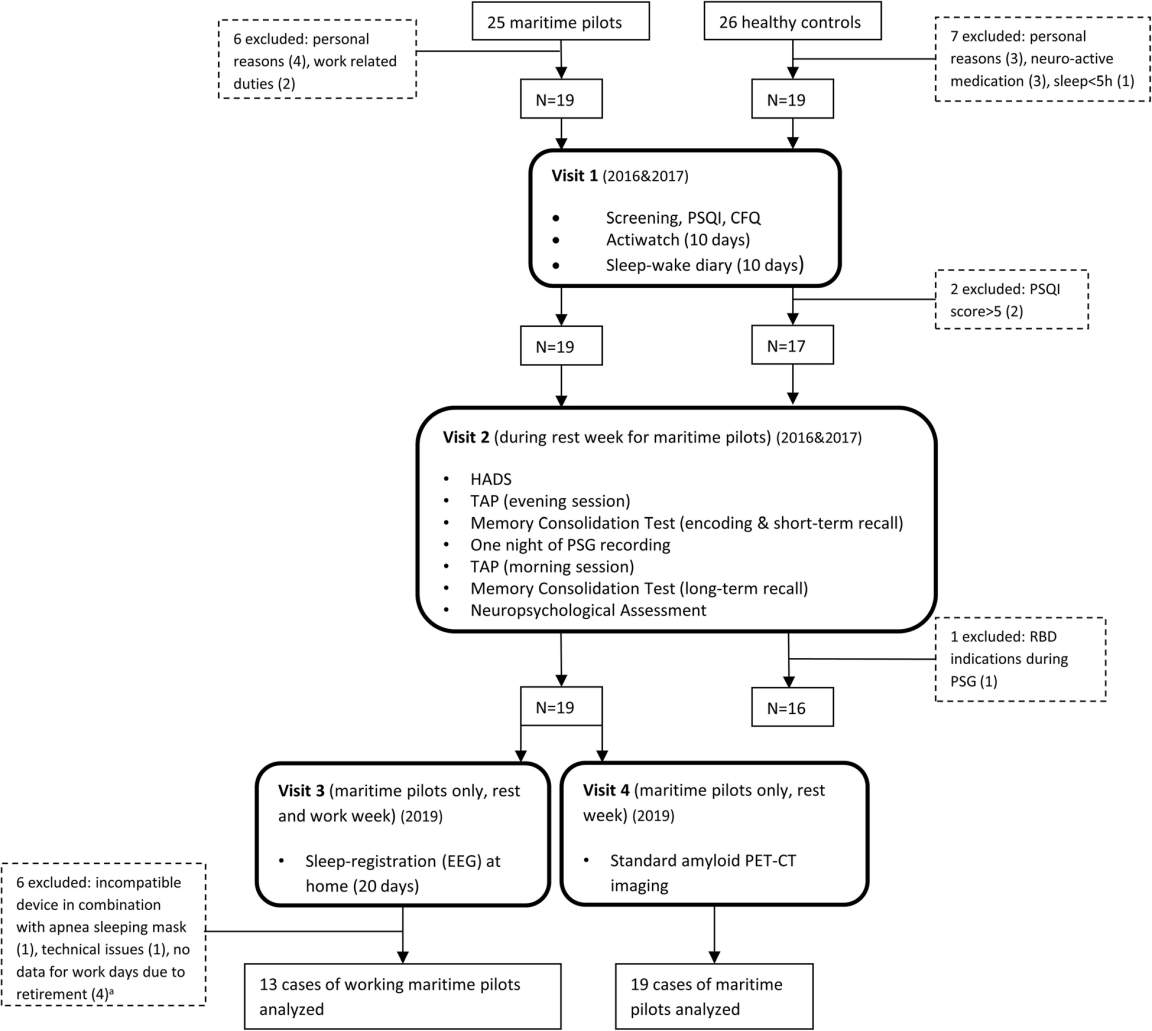
Flow diagram SCHIP study. *Abbreviations:* PSQI, Pittsburgh Sleep Quality Index; CFQ, Cognitive Failure Questionnaire; HADS, Hospital Anxiety and Depression Scale; TAP, Test of Attentional Performance; RBD, REM sleep behavior disorder; PSG, polysomnography. Superscript letter “a” indicates that over the course of the study, four maritime pilots went into retirement; therefore, analysis for the work days is based on the remaining, employed maritime pilots (*n* = 13)

Maritime pilots were compared to age-, sex-, and education-matched healthy volunteers (*n* = 16; mean age = 57; age range 51 to 62 years) with occupations comparable in intellectual demand, but with regular working hours (no shift work). Control participants had normal sleep, confirmed by a Pittsburg Sleep Quality Index (PSQI) of < 5 as well as regular bed times (between 8 p.m. and midnight), and regular wake-up times (between 5 a.m. and 9 a.m.). Participants were excluded from taking part in the study if they were using neuroactive medications, consumed > 30 alcoholic beverages per week, had a body mass index of > 30 kg/m^2^, suffered from intrinsic sleeping disorders (i.e., insomnia, REM sleep behavioral disorder; ruled out by PSG) or, for controls only, if they had self-reported cognitive complaints (indicated by Cognitive Failure Questionnaire (CFQ) and general health questionnaire). Vascular health was assessed during study visits, using a history of health-related events, i.e., high cholesterol, smoking, diabetes, and hypertension in addition to blood pressure measurements in maritime pilots. Baseline characteristics are listed in Table 1; Fig. 1 provides a study flow chart.

### Ethical approval

The SCHIP study was approved by the institutional review board (CMO Region Arnhem-Nijmegen, NL55712.091.16, file number 2016-2337) and performed in accordance with good clinical practice guidelines and the world medical associations code of ethics (Declaration of Helsinki). Written informed consent was obtained from all participants after they received detailed study information. Participants received a stipend of 50 euros for participating, travel costs were compensated.

### Sleep measurements

We combined several complementary methods to assess sleep quality.

#### PSQI

PSQI was filled in twice by maritime pilots (work and rest week) and once by controls.

#### Actigraphy and sleep log

Accelerometer-based sleep measurements (Actiwatch 2; Philips Respironics, Eindhoven, The Netherlands) in maritime pilots and controls were collected for a period of 10 consecutive days. Data was validated with sleep-wake diaries. For maritime pilots, these days contained a mixture of work and rest days. Measurements were taken during the screening phase of the study (Fig. 1), before cognitive testing and amyloid PET scans.

#### Polysomnography (2016/2017)

To ascertain that neither maritime pilots nor controls had intrinsic sleep disorders, we performed full polysomnography (PSG) in a sleep lab (Kempenhaeghe, Heeze, The Netherlands), registering total sleep time (TST), sleep stages (N1, N2, N3, REM), wake time after sleep onset (WASO), sleep efficiency (SEF), and sleep onset latency (SOL). The PSG took place on Sunday nights during rest weeks for maritime pilots and normal weekend days for controls, between 2016 and 2017 (Fig. 1).

#### Home-EEG (2019)

We performed objective measurements of sleep quality during work and rest weeks in 13 of the 19 maritime pilots. For this, we used a novel and innovative device with a dry, single-lead EEG electrode (SmartSleep; Philips, Eindhoven, The Netherlands) [23]. This device is a headband that records EEG signals and can differentiate between light and deep sleep. Furthermore, it measures total sleep time (TST), sleep onset latency (SOL), number of short awakenings (< 5 min awake), and number of arousals (> 5 min awake). Maritime pilots were instructed to wear the headband for 20 consecutive days (10 workdays and 10 rest days). These measurements took place in 2019, before the amyloid PET scan (Fig. 1).

### Cognitive assessment

The aim of the cognitive assessment was to explore effects of long-term exposure to sleep disruption in maritime pilots on AD-related cognitive impairment. Therefore, we applied a cognitive test battery that was designed to detect cognitive dysfunction in preclinical AD [24]. Tests focused on episodic memory (Logical Memory Subtest from the Wechsler Memory Scale – Fourth Edition (WMS-IV LM), Rey Auditory Verbal Learning Test (RAVLT)), semantic memory and language (letter and semantic fluency, Boston Naming Test), working memory and executive function (Digit Span subtest from the Wechsler Adult Intelligence Scale – Fourth Edition (WAIS-IV), Trail Making Test (TMT) parts A and B, WAIS-IV Coding), and attention (Test of Attentional Performance 2.0, TAP). Overnight memory consolidation was assessed using a novel paradigm based on the Doors Test [25], assessing visual recognition memory after short delay (10 min) and memory consolidation after long delay (after sleep). An overview about the cognitive tasks is summarized in the additional information file (see Additional file 2) and described in detail in our methods paper [22]. Cognitive tests were performed in the morning following PSG (in 2016 and 2017).

### Amyloid PET-CT imaging with ^18^F-flutemetamol

Brain PET-CT scans were acquired in 2019 (Fig. 1) in maritime pilots only, since outcomes can be compared to normative data from the literature. We used the validated tracer ^18^F-flutemetamol [26], a tracer that performs comparable to ^11^C-PIB [27]. Previous studies suggested that CSF and PET measurements of amyloid-β are in high concordance [28–31], while some suggest that PET is more powerful and more specific to AD pathology [32, 33]. Static brain images were acquired 90–110 min post-injection (four frames of 5 min) after bolus injection of approximately 185 MBq ^18^F-flutemetamol on a Siemens Biograph mCT. To measure tissue uptake ratios, PET scans of the PET-CT session were registered to the CT scan of the PET-CT session by rigid body linear registration with nearest neighbor interpolation using FSL’s FLIRT (FMRIB’s Linear Image Registration Tool) [34–36]. CT scans were then registered to the MNI152 2mm skull template by affine linear registration with a mutual information cost function and nearest neighbor interpolation using FSL’s FLIRT and by non-linear registration using FSL’s FNIRT (FMRIB’s Non-linear Registration Tool) [34, 35]. These transformations were combined to align the PET scan to the MNI152 space in one single step. Tissue ratio was used as outcome measure, which is equivalent to the standard uptake value ratio (SUVR). The global cortical areas as well as prefrontal and temporal cortex and the cerebellum were selected with the MNI152 2 mm cortical atlas. Subsequently mean uptake values of these regions of interest (ROIs) and the tissue ratio, equivalent to the SUVR, were calculated using the cerebellum as reference region [37, 38]. Normal global SUVR in a cognitively healthy population (aged 30–60) was 1.3 (± 0.09) [27], comparable to mean SUVR of 1.29 (± 0.2) reported by Thurfjell et al. [39]. As additional step, all scans were visually rated as positive/negative for the presence of amyloid-β deposition by an experienced and trained [40] nuclear medicine physician (JB) using validated criteria [37].

In the original protocol, an additional MRI scan was planned for PET-MRI co-registration to allow more detailed regional analyses of amyloid-β uptake [22]. Because of limitations in funding, this could not be performed for this study after all.

### Statistical analysis

Statistical analyses were performed using IBM SPSS Statistics for Windows, version 20.0 (IBM Corp., Armonk, NY, USA). Alpha was set at 0.05 and tested two-sided. All continuous variables were assessed for normal distribution by inspection of histograms and the Shapiro-Wilk test. Normally distributed data are shown as mean ± SD. Not normally distributed data are presented with median and interquartile ranges (IQR). For primary outcomes of cognitive assessment, raw test scores were transformed into *z*-scores for each neuropsychological test. *Z*-scores were computed in SPSS and were based on mean and SD of the whole sample. For the other primary outcome measure, amyloid-β burden, we used the mean global standard value uptake ratio (SUVR) and the dichotomous visual read of the amyloid PET scans (positive/negative) [37]. An independent samples *t*-test was performed to compare normally distributed outcome measures between the groups (shown as mean ± SD); not normally distributed outcome measures were assessed with Mann-Whitney *U* tests (shown as median (IQR)). With regard to the home-EEG data, a Wilcoxon signed rank test was performed to compare deep sleep time (DST) during work weeks to DST during rest periods.

## Results

After exclusion and drop-out of participants (see Fig. 1), 19 maritime pilots and 16 controls completed the study (Fig. 1, Table 1).

**Table 1:** Baseline characteristics

Characteristics	Controls, *n* = 16	Maritime pilots, *n* = 19
Age, years	57 ± 2.9	53 ± 3.4
Educational attainment, years	17.4 ± 7.3	18 ± 0
BMI, kg/m^2^	25.5 ± 2.7	25.7 ± 2.7
History of diabetes	0 (0)	0 (0)
SBP, mmHg	NA	148.0 ± 16.4
DBP, mmHg	NA	90.16 ± 11.7
Medication (“yes/no”)	2 (10.5)	4 (21.1)
Smoking (“yes/no”)	3 (15.8)	3 (15.8)
History of hypertension	0 (0)	0 (0)
History of high cholesterol	0 (0)	1 (5.3)
CFQ	26.4 ± 10.8	29 ± 7.8
HADS Anxiety	4.8 ± 3	4.0 ± 1.7
HADS Depression	3.6 ± 2.5	3.7 ± 2.7

Data is shown as mean ± SD or no. (%) (for normally distributed data)

*Abbreviations*: *BMI* body mass index, *SBP* systolic blood pressure, *DBP* diastolic blood pressure, *CFQ* Cognitive Failure Questionnaire, *HADS* Hospital Anxiety and Depression Scale, *NA* not applicable

Maritime pilots were on average 4 years younger than controls (Table 1; 95% CI − 6.139 to − 1.716). Results from the independent *t*-test did not indicate other differences between the groups at baseline (Table 1). All participants were Dutch, of white European descent, and had the same level of education.

### Sleep characteristics

#### PSQI

Maritime pilots reported worse sleep quality on the PSQI compared to controls, during rest weeks but especially during work weeks (Table 2). When comparing PSQI scores between work week and rest week within maritime pilots, results of the *t*-test revealed that the average PSQI score for work weeks was almost twice the score for rest weeks, with values exceeding the validated cutoff point (≥ 7) for abnormal sleep behavior (Table 2).

#### Actigraphy

Subjective reports (PSQI) of poor sleep was confirmed by data from 10 days of actigraphy (mix of workdays and rest days), which indicated more awakenings and less sleep efficiency in the maritime pilot group compared to controls (Table 2).

**Table 2:** Comprehensive sleep characteristics of maritime pilots and controls

Measures		Controls, *n* = 16	Maritime pilots, *n* = 19	*p* value
PSG	TST, min	406 ± 44	403 ± 51	0.86
	N1, min	46 ± 18	41 ± 14	0.40
	N2, min	232 ± 36	215 ± 36	0.20
	DST, min	50 ± 25	66 ± 28	0.10
	REM, min	68 ± 17	79 ± 17	0.10
	WASO, min	61 ± 26	53 ± 39	0.48
	SEF, %	85.8 ± 7.1	86.1 ± 9.4	0.91
	SOL, min	8 ± 7	11 ± 9	0.32
Actiwatch	No. awakenings	33.5 ± 11.1	37.8 ± 10.3	0.24
	SEF, %	89.3 ± 4.3	86 ± 3.8	0.03*
PSQI (rest week vs. control	Overall score	3.2 ± 1.4	4.5 ± 2.2*	0.049*
PSQI (work week vs. control)	Overall score	3.2 ± 1.4	8.8 ± 2.9**	< 0.001**

Data is shown as mean ± SD (for normally distributed data)

Actiwatch data and PSQI were collected in 2016 and 2017. Actiwatch data was collected for a period of 10 consecutive days; for maritime pilots, these 10 days were a mix of work and rest days. PSQI was administered twice for maritime pilots, including one work week and one rest week

*Abbreviations*: *PSG* polysomnography, *TST* total sleep time, *DST* deep sleep time, *REM* rapid eye movement sleep, *WASO* wake after sleep onset, *SEF* sleep efficiency, *SOL* sleep onset latency, *PSQI* Pittsburgh Sleep Quality Index

*Significant at *p* <0 .05

**Significant at *p* < 0.001

#### PSG (2016/2017)

Both maritime pilots and controls had normal sleep patterns, including normal amount of DST (Table 2), ruling out intrinsic sleep disorders and indicating undisturbed sleep in maritime pilots during rest days.

#### Home-EEG (2019)

**Fig. 2:**
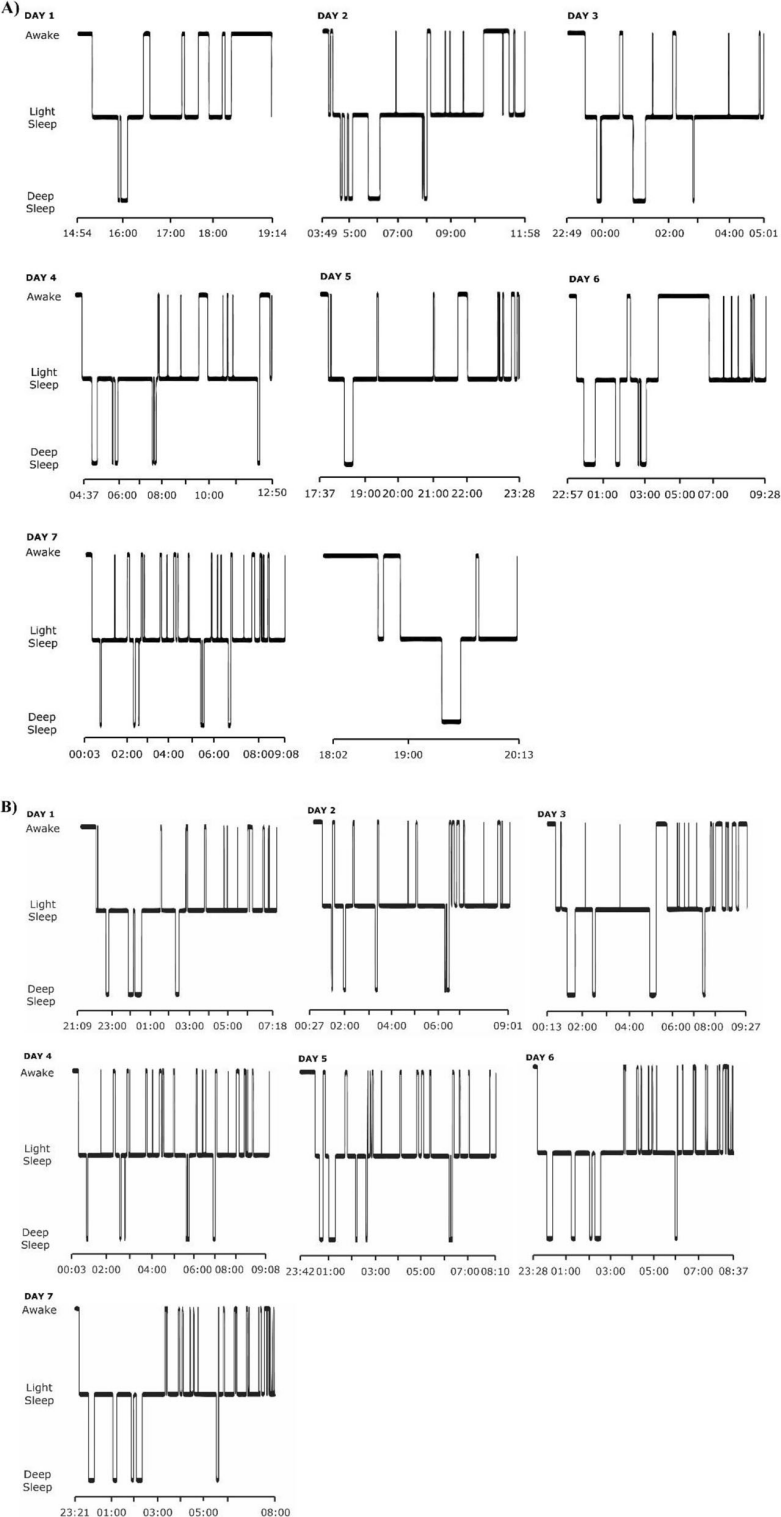
Example of a maritime pilots’ sleep schedule. **a** Hypnogram is based on 7 consecutive working days of sleep measurements with a dry electrode single-lead home-EEG device. **b** Hypnogram is based on 7 rest days of sleep measurements with a dry electrode single-lead home-EEG device

We were able to analyze EEG-based sleep measurements during work and rest days in 13 maritime pilots (of the *n* = 19 maritime pilots, 4 had retired by 2019 when these measurements were scheduled, and could therefore no longer be measured during workdays; 2 could not be analyzed due to technical issues) (Fig. 1). Maritime pilots showed less TST during work weeks compared to rest weeks (*Z* = − 3.18; *p* = 0.001) as well as less DST during work weeks compared to rest weeks (*Z* = − 2.83; *p* = 0.005) (Table 3). Based on the home-EEG measurements, we created hypnograms of one maritime pilot for a work week and a rest week, illustrated in Fig. 2.

**Table 3:** Results from the home-EEG measurements (maritime pilots only)

Measures		Rest week, *n* = 13	Work week, *n* = 13	*p* value
Home EEG	TST^†^, min	406.17 (340–425.98)	318.56 (250.21–352.93)	0.001*
	DST^†^, min	51.34 (48.37–69.30)	36.75 (32.30–58.58)	0.005*

Data is shown as median (IQR) (for not normally distributed data)

Home-EEG recordings were performed in 2019 in maritime pilots only using a dry single-lead EEG device (Philips, Eindhoven, The Netherlands)

*Abbreviations*: *TST* total sleep time, *DST* deep sleep time

*Significant at *p* < 0.05

^†^Means calculated based on sleep periods within work week or rest week respectively

### Cognitive assessment

For cognitive assessment, we transformed all raw neuropsychological test scores into *z*-scores. Results from the independent *t*-test did not indicate differences between maritime pilots and controls on tests of episodic memory (WMS-IV LM recognition, RAVLT total median). Small differences were observed on semantic memory and language, in which maritime pilots performed slightly better on the Boston Naming Test compared to controls. Performance on working memory and executive function (WAIS-IV, TMT, WAIS-IV Coding) and attention (TAP 2.0) did not differ significantly between the groups. Maritime pilots performed slightly better on the visual recognition memory after short delay compared to controls. Long-term memory consolidation, however, did not differ between the groups. All test scores were within normal age- and education-adjusted ranges based on available normative data (data not shown). All results can be found in Table 4.

**Table 4:** Results of cognitive assessment and memory consolidation

Measures		Controls, *n* = 16	Maritime pilots, *n* = 19	*p* value
WMS-IV	LM I	0.16 ± 1.07	− 0.08 ± 0.99	0.49
	LM II	0.29 (− 0.93–1.01)	0.11 (− 0.61–0.83)	0.72
	LM recognition	0.25 ± 1.10	− 0.12 ± 0.94	0.29
RAVLT	Total	− 0.08 (− 0.63–0.61)	0.77 (− 1.14–1.09)	0.41
	Del. recall	− 0.08 ± 0.76	0.06 ± 1.25	0.70
	Del. recognition	− 0.10 ± 1.15	0.21 ± 0.85	0.37
	Sensitivity A’	0.07 (− 0.85–0.82)	0.22 (− 0.30–0.82)	0.41
WAIS-IV	Coding	− 0.10 ± 0.59	0.21 ± 1.26	0.37
	Digit span	− 0.21 ± 0.62	0.23 ± 1.24	0.21
TMT	Part A	− 0.09 ± 0.75	− 0.06 ± 1.15	0.94
	Part B	− 0.33 (− 0.57–0.75)	− 0.38 (− 0.96–0.50)	0.24
Fluency	D-A-T	0.07 ± 0.89	− 0.29 ± 1.13	0.78
	Animal	0.38 (− 0.88–0.73)	0.20 (− 0.70–0.91)	0.84
	Profession	− 0.26 ± 0.85	0.26 ± 1.13	0.14
BNT	Short version	− 0.11 (− 0.39–0.31)	0.20 (0.10–0.62)	0.02*
TAP evening	Cued	− 0.05 (− 0.72–0.44)	− 0.39 (− 0.91–0.50)	0.37
	Un-cued	− 0.05 (− 0.87–0.89)	− 0.17 (− 0.76–0.45)	0.84
TAP morning	Cued	− 0.10 (− 0.50–0.66)	− 0.29 (− 0.71–0.12)	0.27
	Un-cued	− 0.13 (− 0.63–1.09)	− 0.40 (− 0.74–0.33)	0.22
Visual recognition—short delay (10 min)	Sensitivity, A’	− 0.27 ± 0.90	0.46 ± 0.58	0.007*
	Hits	− 0.33 (− 1.18–0.15)	0.64 (0.15–0.64)	0.03*
	False alarms	0.19 ± 1.06	− 0.27 ± 0.84	0.16
Memory consolidation—long delay (after sleep)	Sensitivity, A′	− 0.08 ± 0.95	0.35 ± 0.73	0.14
	Hits	0.50 (− 0.21–0.70)	− 0.08 (− 0.69–0.54)	0.20
	False alarms	0.15 ± 0.90	− 0.41 ± 0.76	0.06

Data is shown as mean ± SD (for normally distributed data) or median (IQR) (for not normally distributed data)

Test results are expressed in *z*-scores. TAP: *z*-scores are based on median reaction-time. Visual recognition—short-term: assessed approximately 10 min after targets were presented. Memory consolidation after long-term took place after one night of sleep (approximately 10 h)

*Abbreviations*: *WMS* Wechsler Memory Scale, *LM* logical memory, *RAVLT* Rey Auditory Verbal Learning Test, *WAIS* Wechsler Adult Intelligent Scale, *TMT* Trail Making test, *BNT* Boston Naming Test, *TAP* Test of Attentional Performance

*Significant at *p* < 0.05

### ^18^F-flutemetamol PET-CT

**Fig. 3:**
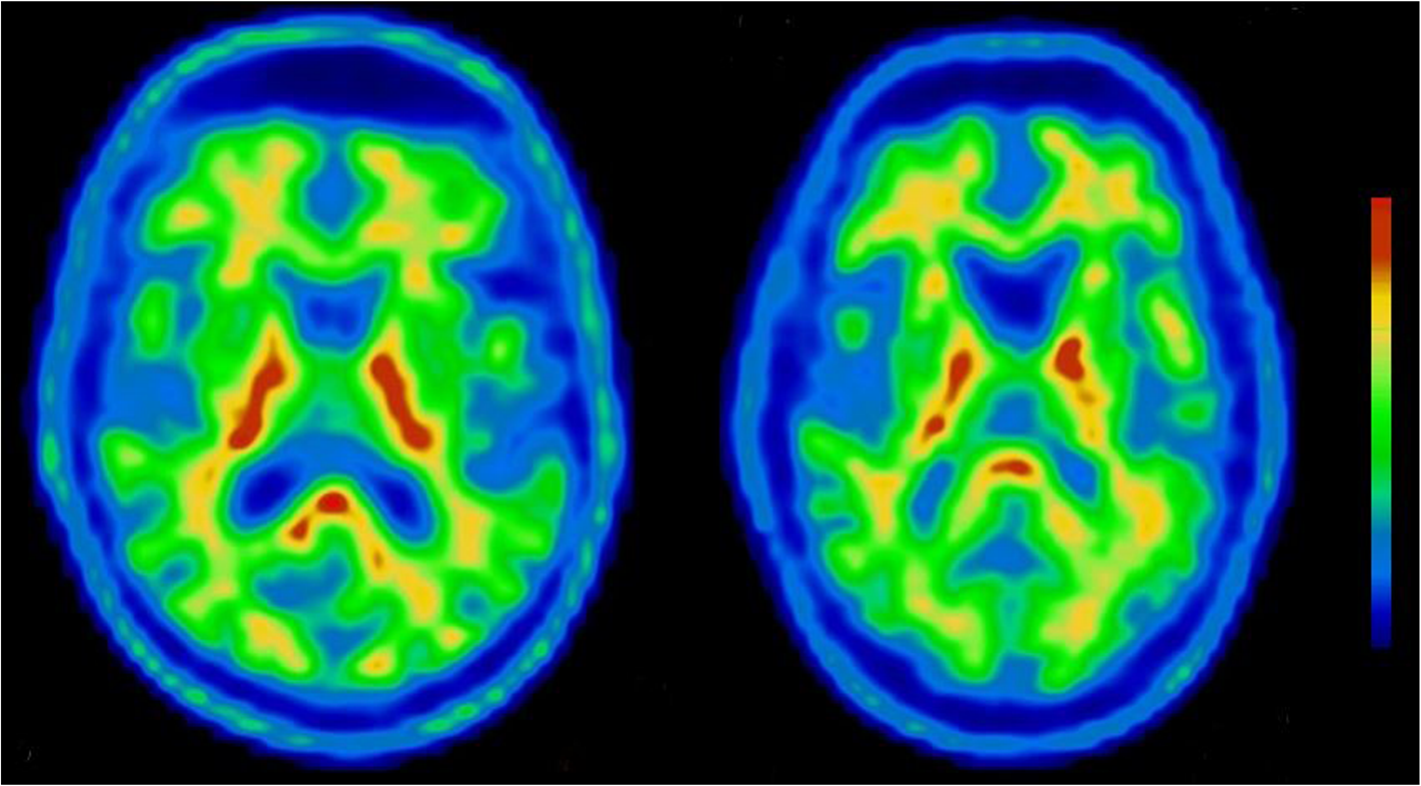
Representative transversal slides from 18F-flutemetamol PET scans of two participants. Scans were acquired 90–110 min post-injection and show normal subcortical nonspecific uptake in the brain

Amyloid PET scans were administered in maritime pilots only (*n* = 19, Fig. 1). SUVRs in healthy populations were reported as 1.29 (± 0.2) [39] and 1.3 (± 0.09) [27]. The global cortical SUVR in maritime pilots was 1.009 (± 0.059; 95% CI 0.980 to 1.037) and therefore below normal values for a cognitively healthy population in this age range [27, 39]. More specifically, we detected a SUVR of 0.860 (± 0.098; 95% CI 0.813 to 0.907) for frontal lobes and a SUVR of 0.996 (± 0.06; 95% CI 0.967 to 1.025) for temporal lobes. In addition, all scans were rated negative for the presence of amyloid-β deposition on visual reading. Figure 3 shows examples of amyloid PET images from two representative participants. There were no correlations between SUVRs and sleep quality (PSQI overall score and DST (for rest and work weeks)).

## Discussion

We investigated global brain amyloid-β levels and cognitive function in a unique population experiencing long-term sleep disruption, wherein every other week was characterized by sleep disruption due to irregular working hours.

Our main finding is that, in this relatively small, but deeply phenotyped sample, this intensity and pattern of sleep disruption was not associated with elevated brain amyloid-β levels, nor with cognitive decline.

In previous studies, a single night of full sleep deprivation, or selective restriction of deep sleep, and chronic partial sleep fragmentation (rodents only) increased brain amyloid-β levels [8–13]. These observations have fueled the hypothesis that repeated nights of sleep loss may contribute to the risk of dementia due to AD by gradually increasing amyloid-β levels.

The sample of maritime pilots offered a unique opportunity to explore if long-term, externally induced sleep disruptions increase dementia risk in terms of AD-related impaired cognitive function and amyloid-β burden. Their sleep behavior is characterized by work weeks with disrupted sleep, alternating with rest weeks of unrestricted sleep. This pattern was confirmed using a combination of methods: self-reported disrupted sleep during work weeks was objectified by sleep diaries, actigraphy, and home-EEG measurements. Relatively normal sleep during rest weeks of maritime pilots was furthermore confirmed with PSG (compared to controls) and home-EEG measurements. Moreover, using PSG we were able to exclude intrinsic sleep disorders in this group, which is important because sleep loss may be an early manifestation of Alzheimer’s pathology and could lead to a reverse causality association [41, 42]. To explore possible AD-related impaired cognitive performance, we applied a cognitive test battery that was chosen for its sensitivity to cognitive changes in early, preclinical AD [24]. On all cognitive domains, maritime pilots showed normal cognitive performance, compared not only to the control group, but also to normative values. This was also the case for overnight episodic memory consolidation, which is dependent on deep sleep [5, 43].

We considered that normal cognitive function would not rule out increased amyloid-β levels, since early stages of amyloid-β accumulation (indicated by PET or CSF) can have a long asymptomatic stage. Therefore, we performed additional global brain amyloid-β imaging in maritime pilots. None of the maritime pilots had evidence of elevated amyloid-β levels, with SUVR values remaining below the values established for a healthy population [27, 39]. In a recent meta-analysis, the estimated prevalence of PET amyloid-positivity in cognitively healthy men aged 55–60 years was 13% (95% CI 10.3 to 16%) [44]. This indicates that our observation of a prevalence of 0/19 confidently rules out elevated amyloid-β levels, even considering the relatively small sample size.

What could explain the observed absence of elevated amyloid-β levels or impaired cognitive function, despite evidence of long-term sleep disruptions?

First, assuming that the hypothesis that sleep disruption may cause AD is correct, the alternating pattern of a week with unrestricted sleep following a week of disrupted sleep may be insufficient to cause elevated brain amyloid-β levels. Either sleep disruption during ≈ 50% of nights for ≈ 20 years is insufficient to affect amyloid-β clearance/production or the week of normal sleep following a week of sleep disruption provides compensatory reductions in brain amyloid-β levels. This latter option would then suggest that disrupted sleep is a modifiable risk factor and that it may not be necessary to achieve full normalization of sleep to reduce AD risk. Whether this can be extrapolated to the general population is uncertain however. The maritime pilots may, due to their profession, be better able to compensate normal sleep in their rest weeks. While most studies link reduced total sleep time (< 6 h) to increased AD risk [1, 20], other work suggests that the risk is specifically linked to reduced deep sleep [12, 17]. The maritime pilots had reduced total sleep time during work weeks, but the home-EEG recordings indicate that they still achieved an average of 37 min of deep sleep per sleep period. Therefore, SWS may have been insufficiently impaired to result in abnormal amyloid-β levels. This argument is, however, not supported by recent work demonstrating that a reduction in total sleep time, but not SWS, determined the increase in amyloid-β production [20].

Second, it is possible that sleep disruption alone is insufficient to increase AD risk, but requires the presence of other risk factors, such as impaired glucose metabolism [45], oxidative stress [46], depression [47], or general poor vascular health [48]. Our study population was healthy and had a low vascular risk (Table 1).

Current research on this topic is still in its very early stage, with limited evidence supporting a causal relationship between sleep loss and risk of AD dementia. The association between sleep loss and AD may be driven by reverse causality (sleep loss as an early manifestation of AD) or by a shared common pathway that causes sleep loss and increases AD risk. There is also recent evidence that found no association between sleep (subjective sleep quality) and risk of dementia [49].

Previous evidence suggesting a link between sleep and AD has been limited to a small number of studies in rodents and humans, with variations in methodology and study population selection. Furthermore, the human studies focused on the relationship between poor sleep for a short period of time (1 or 2 nights) and its effects on amyloid-β (or tau), but have not studied actual development of AD dementia.

Longitudinal studies are available but lack rigorous assessment of sleep and biomarker evidence of AD. Our study adds information on the long-term association between poor sleep and AD, combining objective sleep measures with established biomarkers for AD.

### Strengths and limitations

A strength of the study is the comprehensive assessments of all outcome measures: cognitive function was assessed with an extensive test battery sensitive to early, preclinical symptoms of AD; sleep was assessed with various measurements including self-reported but also objectively measured sleep, implementing innovative techniques for sleep assessments (home-EEG); sleep disorders were ruled out using PSG; and PET-amyloid imaging was used as a validated AD biomarker. A further strength is the unique cohort of maritime pilots, with prolonged and consistent exposure to sleep loss related to their work, making this a highly valuable population that allowed us to explore poor sleep as isolated variable in relationship with the risk of AD dementia.

Our study is limited by the small sample size. Home-EEG measurements were available in 13 of the 19 maritime pilots. However, outcomes of these sleep measurements confirmed observations of work-related disrupted sleep based on PSQI, sleep-wake dairy and actigraphy data in the whole sample, and added novel data on total and deep sleep time during work weeks and rest weeks.

The uniqueness of the population may also cause bias. Maritime pilots are healthy, have no cardiovascular risk, and are physically active in their work, factors that may reduce their AD risk. They may be resilient to the consequences of sleep disruption, because they have successfully performed this work for > 10 years. One example of such resilience could be their ability to achieve deep sleep even under conditions of fragmented and restricted total sleep during work weeks or their capacity to generate sufficient deep sleep during rest weeks.

Another limitation is that controls, although matched for sex, age, education, and general health, might not have been matched entirely with regard to personality, resilience, physical activity, or cognitive skills/general intelligence.

One could argue that the absence of tau measurements is a limitation, as recent evidence now also suggests that sleep affects tau in a similar manner as amyloid-β [50]. We did not perform tau measurements because the maritime pilots had no evidence of cognitive impairment. Since tau pathology is strongly correlated with cognitive decline [51–53], it is highly unlikely to find evidence of tau accumulation in subjects with normal cognitive function, even more so when they are amyloid-negative. A final limitation is that amyloid-β status was not obtained from the controls. Instead, we compared our outcomes to normative values from the literature, which were acquired with additional MRI measurements for co-registration of the amyloid PET-CT scans. Since we used CT to identify global amyloid-β instead of MRI, this difference in methodology has to be kept in mind when interpreting our results.

## Conclusions

In this study, we tested the hypothesis that prolonged sleep loss increases the risk of dementia due to AD. We found that a history of work-induced, long-term sleep disruption was not associated with impairment in cognitive function, nor with elevated global brain amyloid-β levels, in a group of healthy, middle-aged men. Taking into account the small sample size of our study, our results do not necessarily refute the hypothesis we intended to test, but neither support it. It is possible that amyloid-β accumulation during periods with sleep disruption can be reduced in nights with normal sleep. Alternatively, sleep loss may only increase AD risk in combination with other factors. Finally, the association between sleep loss and AD in epidemiological studies may be driven by reverse causality. These and other hypotheses have to be tested in future studies.

## Supplementary information

**Additional file 1.** Details of the study population and their occupation.

**Additional file 2. Supplemental Box 1:** Overview Neuropsychological Test Battery.

## Data Availability

Data generated and/or analyzed during this study are included in this article and its supplementary information files. Additionally, the datasets used/analyzed in the current study are available from the corresponding author on reasonable request.

## Abbreviations

AD: Alzheimer’s disease
CFQ: Cognitive Failure Questionnaire
DBP: Diastolic blood pressure
DST: Deep sleep time
HADS: Hospital Anxiety and Depression Scale
IQR: Interquartile range
NA: Not applicable
NRA: Number of arousals
NRI: Number of interruptions
PSG: Polysomnography
PSQI: Pittsburgh Sleep Quality Index
RAVLT: Rey Auditory Verbal Learning Test
REM: Rapid eye movement
ROI: Region of interest
SBP: Systolic blood pressure
SCHIP: Sleep Cognition Hypothesis In maritime Pilots
SD: Standard deviation
SEF: Sleep efficiency
SOL: Sleep onset latency
SUVR: Standard uptake value ratio
SWS: Slow wave sleep
TAP: Test of Attentional Performance
TMT: Trail Making Test
TST: Total sleep time
WAIS-IV: Wechsler Adult Intelligence Scale (4th edition)
WASO: Wake after sleep onset
WMS-IV LM: Wechsler Memory Scale (4th edition) Logical Memory
