# Supplementary material for: Effects of long-term sleep disruption on cognitive function and brain amyloid-β burden: a case-control study
Source: Alzheimers Res Ther. 2020 Aug 26;12:101. doi: 10.1186/s13195-020-00668-5 (PMC7450576; doi:10.1186/s13195-020-00668-5)
Supplement: Supplementary file 1 — Additional file 1. Details of the study population and their occupation. [file 13195_2020_668_MOESM1_ESM.docx]

**ADDITIONAL FILE 1**

**Details of the study population and their occupation**

Maritime pilots from the national organization of Dutch Maritime Pilots (Nederlandse Loodswezen) work in irregular und unpredictable shift-work schedules. The responsibility of a maritime pilot is to handle large international ships arriving by sea and to maneuver them into their final docking position in one of the Dutch harbors. Their work amount is determined by the number and kind of ships that arrive. Once a ship has arrived, one maritime pilot is sent out from the harbor to the ship, that is usually still many kilometers away from the coast line. Once arrived, the maritime pilot boards the ship using a ship ladder attached to the hull of the ship, and takes over from the captain. Their task then is to navigate the ship and maneuver it into the final docking position in one of the Dutch harbors. This profession demands high responsibilities and requires precise knowledge of the dimensions of the harbor and the ships besides technical- and navigational skills. Furthermore, communication and social skills additionally to good English proficiency is of advantage in communication with the international crew on board. Guiding the ships is a time intensive procedure, that can take hours to complete.

In a workweek (7 days) the maritime pilots have to be accessible 24 hours per day during which they can be called several times, depending on the number of ships that arrive. This schedule leads to multiple divided short sleep periods and/or shorter general total sleep time over a period of 24 hours during a workweek. The workweek is followed by a week off with unrestricted sleep.

Maintaining this schedule for many years results in chronic exposure to sleep disruption, either due to partial sleep deprivation (missing a full night of sleep due to work), sleep restriction (a much shorter night of sleep than normal) or sleep fragmentation or disruption (short periods of sleep interrupted by calls to work).
