# Supplementary material for: Effects of long-term sleep disruption on cognitive function and brain amyloid-β burden: a case-control study
Source: Alzheimers Res Ther. 2020 Aug 26;12:101. doi: 10.1186/s13195-020-00668-5 (PMC7450576; doi:10.1186/s13195-020-00668-5)
Supplement: Supplementary file 2 — Additional file 2. Supplemental Box 1: Overview Neuropsychological Test Battery. [file 13195_2020_668_MOESM2_ESM.docx]

**ADDITIONAL FILE 2**

**Supplemental Box 1.** Overview Neuropsychological Test Battery.

| **Cognitive domains** | **Administered test** |
| --- | --- |
| Episodic Memory | WMS-IV Logical Memory  Rey-Auditory Verbal Learning Test (RAVLT) |
| Working Memory/ Executive Functioning | WAIS-IV Digit Span  Trail Making Test (A,B)  WAIS-IV Digit Symbol |
| Semantic Memory/ Language | Letter Fluency (D-A-T)  Semantic Fluency (animal/profession naming)  Boston Naming Test (BNT-short form) |
| Attention | Test of Attentional Performance (TAP 2.3) – evening and morning sessions |
| Visual Recognition | Adjusted version of the Visual Recognition Memory Doors Test (short term recognition and overnight memory consolidation) |
